# Motor Interconnections Between Superior and Inferior Laryngeal Nerves

**DOI:** 10.7759/cureus.2337

**Published:** 2018-03-16

**Authors:** Emin Gurleyik

**Affiliations:** 1 Department of Surgery, Duzce University Medical Faculty

**Keywords:** thyroid, goiter, surgery, ionm, ebsln, rln

## Abstract

Introduction

Anatomical studies on human cadavers have established anastomoses between laryngeal nerves. However, we need to functionally identify motor communication via these anastomoses between the recurrent laryngeal nerve (RLN) and the external branch of the superior laryngeal nerve (EBSLN) in living bodies. We aim to establish motor interconnections using intraoperative nerve monitoring (IONM).

Methods

IONM of 112 EBSLNs and RLNs in 62 thyroidectomy cases was used to establish motor functions of laryngeal nerves. Electrophysiological parameters were recorded, and cricothyroid muscle (CTM) contraction was observed after stimulation of laryngeal nerves.

Results

Eighty (71.4%) EBSLNs were visually identified, and 109 (97.3%) EBSLNs were functionally identified with CTM contraction. Stimulation of 74 (67.9%) EBSLNs induced contraction of laryngeal muscles and generated wave amplitude from intrinsic laryngeal musculature. The stimulation of the RLN induced CTM contraction in 65 (58%) of the 112 muscles. The mean conductivity powers of the EBSLN and of the RLN to intrinsic laryngeal musculature were calculated as 231.3 µV and 1354.5 µV, respectively.

Conclusion

Recordable waveform amplitude with EBSLN stimulation yielded motor relations between laryngeal nerves. CTM contraction after stimulation of the RLN confirmed these relations. These results of IONM established motor interconnections between superior and inferior laryngeal nerves in the majority of patients. The EBSLN may have an effect on motor innervations for intrinsic laryngeal muscles via motor interconnections.

## Introduction

Safer thyroid surgery is based mainly on the anatomy of both the external branch of the superior laryngeal nerve (EBSLN) and the recurrent laryngeal nerve (RLN). Functional integrity of these two components innervating the laryngeal muscular system is of paramount importance for complication-free thyroid surgery. The EBSLN contains motor fibers and innervates the cricothyroid muscle (CTM) that is important in adjusting both the tension and length of the vocal cords (VC). The RLN innervates intrinsic laryngeal musculature that provides all essential movements of the VCs [1-4]. Visual identification, exposure, and preservation of anatomical integrity of the RLN are mandatory for safer surgery. Besides the RLN, the EBSLN is also identified in a considerable number of patients [5,6]. On the other hand, visual integrity of nerve branches does not necessarily guarantee proper motor function.

Intraoperative nerve monitoring (IONM) is used as an adjunct for visual identification to confirm neural motor activity. The stimulation of the RLN creates VC movement that establishes functional integrity of the nerve during thyroid surgery. Electrophysiological stimulation of the EBSLN generates the CTM twitch to establish proper motor activity [1,7-12].

Previous anatomical studies have reported many anastomoses between the EBSLN and the RLN [13-16]. Some interconnections are sensory, while others are motor, and are important for laryngeal musculature. Results of anatomic studies are static findings and are inconclusive to explain the real time motor role of these anastomoses on laryngeal muscles. Based on the motor communicating branches between the two nerves, we hypothesize that stimulation of both nerves may create variable wave amplitudes and also contraction of laryngeal muscles. During thyroid surgery, IONM of both nerves, assessing their real time functions, serves also to establish motor relations between superior and inferior neural systems. Therefore, contraction of intrinsic laryngeal muscles after stimulation of the EBSLN, which creates recordable waveform amplitude, may reveal these relations. On the other side, contraction of the CTM following stimulation of the RLN may also confirm communications of the two nerves.

In this prospective study, we aim to determine the roles of the RLN and the EBSLN on the activity of laryngeal musculature using IONM during thyroid surgery. We tried to record electronic wave amplitudes after electrophysiological stimulation of both the superior and the inferior laryngeal nerves to establish motor interconnections between the two nerve systems. We also tried to visually observe contraction of the CTM induced by the EBSLN and the RLN stimulation.

## Materials and methods

We conducted this prospective study on 62 (48 women and 14 men) consecutive thyroidectomy cases who underwent primary thyroid surgery without lymph node dissection between January and September 2017. The average age of the patients was 49.9 (22-75) years. In these patients, IONM of both the EBSLN and the RLN was used. At the end of surgery, post-dissection motor activity of both laryngeal nerves was monitored to measure transmission power of the electrophysiological stimulus to the laryngeal musculature to ensure functional integrity. The presence of CTM contractions were also visually observed after stimulation of both superior and inferior nerves.

IONM 

Intraoperative nerve monitoring of laryngeal nerves was performed using a nerve integrity monitor (NIM-Response 3.0 System; Medtronic Xomed, Jacksonville, FL, USA). The setup of the device included a stimulation intensity of 1 mA and an amplitude threshold of 100 µV. The nerve integrity monitor was connected to surface electrodes integrated with an endotracheal tube 7.0 or 8.0 (NIM® EMG standard reinforced endotracheal tube; Medtronic Xomed, Jacksonville, FL, USA), which was inserted between the VCs under direct vision during intubation.

EBSLN dissection and monitoring

The sternothyroid muscle was retracted superiorly and laterally. After lateral and caudal retraction of the upper pole of the thyroid, a dissection window was opened between the gland and the inferior constrictor muscle. Upper thyroid vessels were ligated closer to the glandular tissue in the sternothyroid–laryngeal triangle under the guidance of nerve monitoring. During and after full mobilization of the upper pole, we tried to identify the EBSLN visually and to check its motor function by IONM in the triangle and on the constrictor muscle. The rate of both visual and functional identification of the EBSLN was determined at the end of the lobe dissection. The two steps of EBSLN monitoring were as follows:

             S1: stimulation of the EBSLN when first identified and during upper pole dissection.

             S2: stimulation of the EBSLN at the end of upper pole dissection and at the end of surgery [17].

The dissection plane between the upper pole of the thyroid and the inferior constrictor muscle was carefully observed to identify the EBSLN visually, and then functional identification was performed using IONM before ligation of vascular branches (S1). After full mobilization of the upper pole, the functional integrity of the EBSLN was re-checked by IONM (S2). The nerve branch was stimulated at a point of 2-3 cm from the edge of CTM. The stimulator probe was applied directly on the EBSLN if it was visualized. If the nerve was not clearly visualized, the nerve tract on the constrictor muscle was stimulated indirectly to create and observe the physiological response of the CTM whose muscular twitch was observed macroscopically.

Stimulation of the EBSLN generated contraction of laryngeal muscles perceived with a sound signal while recording the electromyographic wave amplitude in µV. The latency was also measured and recorded in millisecond (mS). Contraction of the CTM represented functional integrity of the EBSLN.

RLN dissection and monitoring

After medial mobilization of the bilateral lobes of the thyroid gland, the RLN was identified and isolated fully using a conventional lateral approach. When first identified, and after complete exposure under direct vision, the RLN was stimulated and provided conduction of stimulating electricity to the innervated musculature. The IONM was performed as a four-step procedure [18,19]:

V1: stimulation of the vagus nerve (VN) before dissection of the thyroid lobe.

R1: stimulation of the RLN when first identified at the tracheoesophageal groove.

R2: stimulation of the RLN after complete dissection of the lateral thyroid lobe.

V2: stimulation of the VN after complete dissection of the lateral thyroid lobe.

Intraoperatively, the sound signal of motor electrophysiological activity was obtained from the device while the wave amplitude was both measured and recorded. The latency was also measured and recorded in mS. The sound signal and electronic wave amplitude (in µV) indicated the proper functional anatomy of the nerve branches.

We studied the following aspects:

- Rates of visual and functional identification of both the RLN and the EBSLN.

- Results of IONM, such as recorded wave amplitudes and the latency after electrophysiological stimulation of laryngeal nerves.

- Rates of the functional integrity of the RLN with movements of VCs established by IONM, and of the EBSLN with observation of the CTM twitch.

- Rates of contraction of laryngeal muscles induced by EBSLN stimulation established by IONM, and of observation of CTM contraction generated by RLN stimulation.

The effect of the EBSLN on the function of intrinsic laryngeal muscles and the recordable waveform amplitude and latency after stimulation (S2) of the EBSLN were determined at the end of thyroid surgery. Simultaneously, the contraction of the CTM was also observed after stimulation of the RLN. 

Assessment of VC function was performed in all patients who underwent thyroid surgery using preoperative and postoperative laryngoscopy.

## Results

Fifty total thyroidectomies and 12 hemithyroidectomies were performed on these patients. We tried to identify and expose 112 nerves at risk during thyroid surgery (Table 1).

**Table 1:** Thyroid surgery and nerves at risk

	Total thyroidectomy	Right hemithyroidectomy	Left hemithyroidectomy	Total
Patients	50	7	5	62
Nerves at risk	100	7	5	112
Right nerves				57
Left nerves				55

A total of 80 (71.4%) EBSLNs were visually and functionally identified on the inferior constrictor muscle. Using IONM, 109 (97.3%) nerve branches were functionally identified and monitored, the electrophysiological stimulation of which generated CTM twitches. Three EBSLNs on the right side could not be identified either visually or functionally during thyroid surgery (Table 2). All 112 RLNs were identified visually, and their motor functions were confirmed by IONM. At the end of surgery, IONM with a positive response (CTM twitch) reestablished the functional integrity of 109 (97.3%) of 112 EBSLNs. Stimulation of all 112 RLNs (R2) created recordable wave amplitudes that established the functional integrity of RLNs. The stimulation of the RLN generated contraction of the CTM that was visually observed in 65 (58%) of the 112 muscles (Table 3).

**Table 2:** Nerve monitoring and identification rate of the EBSLN *EBSLN: External branch of superior laryngeal nerve. CTM: Cricothyroid muscle. IONM: Intraoperative neuromonitoring.

	Right EBSLN	Left EBSLN	Total
	n (%)	n (%)	n (%)
EBSLN* identified by IONM* with CTM* twitch	54 (94.8)	55 (100)	109 (97.3)
Visual identification rate of the EBSLN	42 (73.7)	38 (69.1)	80 (71.4)
Contribution of IONM to the EBSLN identification	12 (21.1)	17 (30.9)	29 (25.9)
Unidentified EBSLN	3 (5.2)	----	3 (2.7)
Total	57 (100)	55 (100)	112 (100)

**Table 3:** EBSLN stimulation inducing laryngeal muscles contraction and RLN stimulation generating CTM contraction *EBSLN: External branch of superior laryngeal nerve. RLN: Recurrent laryngeal nerve. CTM: Cricothyroid muscle. IONM: Intraoperative neuromonitoring.

	Right	Left	Total
	n (%)	n (%)	n (%)
Number of EBSLN* identified by IONM*	54 (94.8)	55 (100)	109 (97.3)
Number of EBSLN stimulation generating recordable wave amplitude	37 (68.5)	37 (67.3)	74 (67.9)
Visual and functional identification of RLN*	57 (100)	55 (100)	112 (100)
Number of RLN stimulation generating CTM* contraction	36 (63.2)	29 (52.7)	65 (58)

At the end of surgery, synchronous stimulation of 109 EBSLNs (S2) and 112 RLNs (R2) established the functional integrity of identified nerves. In addition to CTM twitches, electrophysiological stimulation of 74 (67.9%) EBSLNs generated wave amplitude of more than 100 µV. The mean conductivity powers of the EBSLN and of the RLN to intrinsic laryngeal musculature were calculated as 231.3 µV and 1354.5 µV, respectively.

Normal movements of the VCs were observed in all our patients by postoperative laryngoscopy.

## Discussion

The EBSLN supplies motor innervation to the CTM whose function is to adjust the tension of the VCs. The EBSLN is not always identified visually during thyroid surgery. For example, Lenquist et al. [5] reported 20% of EBSLNs run distally through the pharyngeal constrictor muscle while Friedman et al. [6] reported 15% of EBSLNs were not identified visually. Therefore, identification of some EBSLNs necessitates intramuscular dissection, but dissecting into the pharyngeal constrictor muscle appears to be inadvisable [5]. In this situation we have opportunity to functionally identify non-visual branches. Based on our results, nerve monitoring may increase the identification rate of the EBSLN and enable the confirmation of its motor activity by generating a CTM twitch. When used as an adjunct, IONM may establish appropriate motor functions of laryngeal nerves. During thyroid surgery, we observed that stimulation of some EBSLNs also generates wave amplitude recorded via the surface electrodes of endotracheal tube inserted between the VCs. In addition to innervating the CTM, we studied prospectively the role of the EBSLN on the function of the intrinsic laryngeal musculature using IONM. We also tried to establish the relationships and motor interconnections of both the superior and the inferior laryngeal neural systems.

Our results showed that the majority of EBSLNs (71.4%) could be identified visually after careful dissection of the sternothyroid–laryngeal triangle closer to the thyroid tissue. Previous studies have reported that the EBSLN could be identified visually in up to 90% of thyroidectomy cases [8,13,20-23]. Despite visual integrity, it is not always possible to confirm adequate motor function of the nerve branch. After electrophysiological stimulation of the EBSLN, the CTM twitch is monitored to assess motor integrity. The stimulation of 109 (97.3%) branches generated the CTM twitch, thereby confirming the functional integrity of the EBSLN. Previous studies have reported identification rates of the EBSLN between 65% and 100% using IONM [8,20-24]. The use of IONM enabled a 25.9% increase in our visual identification rate, thereby revealing the significant contribution of nerve monitoring to the identification of the EBSLN. Authors have previously reported the rates of IONM contribution up to 50% in addition to the rate of visual identification [8,21,22,25]. Thanks to electrophysiological stimulation, the identification rate has increased considerably, and the functional integrity of the EBSLN can be determined for appropriate CTM function in the vast majority of patients. In our study, 2.7% of EBSLNs were neither visually nor functionally identified. Patnaik et al. [26] studied the courses of the EBSLN in relation to the inferior constrictor muscle in 29 fresh cadavers. They reported that 16% of nerve branches had Friedman’s type 3 courses and concluded that the EBSLN would not be encountered in a certain percentage of individuals since it lies under the inferior constrictor. In the Bellantone et al. [27] series, the EBSLN was not clearly identified in 11.6% of cases. Therefore, the surgeons could not visually identify the EBSLNs covered by muscle fibers in some patients undergoing thyroidectomy. On the other hand, we can functionally identify considerable percentages of these covered and hidden branches by nerve monitoring.

In our study, recording the wave amplitude in 67.9% of functionally identified nerve branches revealed that stimulation of some EBSLNs provided activity of intrinsic laryngeal muscles. We calculated the average wave amplitude as 231.3 µV after stimulation of the EBSLN. Randolph [28] reported that electromyography (EMG) data could be obtained in 77% of patients, and the average SLN waveform amplitude was 269 µV. In addition, Barczynski et al. [22] obtained a mean wave amplitude of 249.5 µV in 73.9% of identified nerves while Dionigi et al. [17] reported average amplitudes between 259 and 371 µV. Stimulation of the EBSLN generated an EMG in the ipsilateral VCs with mean amplitude of 156.73 µV [13].

What is the pathway of waveform amplitudes generated by stimulation of the EBSLN? This question leads us to think about the connections between the EBSLN and the RLN and the motor relationship between the superior and the inferior laryngeal nerves. Anatomical studies found communication between the two laryngeal nerves [14]. When dissecting the hemilarynges, a neural connection was found exiting the medial surface of the CTM and then entering into the lateral surface of the thyroarytenoid muscle, the human communicating nerve (HCN) [15]. Microdissection of 90 larynges obtained from necropsies established some form of anastomoses between the laryngeal nerves. An anastomosis between the external laryngeal and recurrent nerves was present in 68% of cases as a connecting branch throughout the CTM [16]. The HCN has been reported to be present in 70% of humans [28]. In our series, 74 (67.9%) EBSLNs inducing intralaryngeal muscles contraction with recordable waveform amplitudes may confirm motor interconnection via the HCN. Complementary to the movements of the VCs induced by EBSLN stimulation, authors also reported contraction of the CTM induced by RLN stimulation [14,29].

We first investigated the motor interconnections by stimulating the EBSLN and recorded waveform amplitudes with stimulation of 67.9% of nerve branches. In addition, simultaneously we looked for CTM twitches by stimulating the RLN. Our observation of visual CTM contraction after stimulation of the RLN in 58% of monitored nerves yielded interconnection between superior and inferior nerves. Similarly, in the series by Miyauchi et al. [29], following stimulation of the 51 (73%) ipsilateral RLNs, CTM contractions, recordable amplitudes, or both were observed as another confirmation of motor communication between the superior and the inferior laryngeal systems. Martin-Oviedo et al. [30] observed response from the CTM in seven of 13 (53.8%) patients after the RLN stimulation. Masuoka et al. [14] have reported that the RLN stimulation induced CTM contraction in 36 (51%) of 70 CTMs. The HCN can conduct neural stimulation from the EBSLN to intrinsic laryngeal muscles and also from the RLN to the CTM. Therefore, laryngeal muscles contraction after EBSLN stimulation, and CTM contraction after RLN stimulation confirms that the RLN and SLN systems may have motor interconnections through some neural anastomoses, mainly the HCN.

In our series, nerve monitoring at the end of surgery ensured and confirmed the motor integrity of RLNs. Our findings of recordable waveform amplitudes following EBSLN stimulation revealed an apparent role of the EBSLN in the innervation of the intrinsic laryngeal musculature. A second source of motor innervation to the thyroarytenoid muscle, other than the RLN, has been suggested by both clinical and experimental observations [14,15,17,22,28,29]. These results increase the significance of motor integrity of the EBSLN. We believe in addition to its primary roles in both CTM function and proper VC tension, the EBSLN serves as a second, auxiliary motor source of the intrinsic laryngeal musculature in a considerable number of patients.

## Conclusions

Electrophysiological nerve monitoring ensures the motor integrity of both the EBSLN and the RLN. Functional identification of the EBSLN using IONM makes a considerable contribution to anatomical observations. The CTM twitch after electrophysiological stimulation provides reliable evidence for the functional integrity of the EBSLN. A considerable number of EBSLN stimulations induce contraction of laryngeal muscles via RLN–SLN interconnections and create recordable waveform amplitudes. Visual CTM contractions were also observed after stimulation of the RLN as another confirmation of motor communication between the two nerves.
